# Co-Immunoprecipitation-Coupled Mass Spectrometry Analysis of Zyxin’s Interactome and Phosphosites in Early *Xenopus laevis* Development

**DOI:** 10.3390/ijms27020738

**Published:** 2026-01-11

**Authors:** Elena A. Parshina, Rustam H. Ziganshin, Andrey G. Zaraisky, Natalia Y. Martynova

**Affiliations:** Shemyakin-Ovchinnikov Institute of Bioorganic Chemistry, Russian Academy of Sciences, 117997 Moscow, Russia; lena_parshina5@mail.ru (E.A.P.); ziganshin@mail.ru (R.H.Z.); azaraisky@yahoo.com (A.G.Z.)

**Keywords:** Zyxin, cytoskeleton, focal adhesion, phosphosites, interactome, mass spectrometry, DDA, DIA

## Abstract

Protein complexes, assembled by scaffold proteins, act as molecular machines driving development. The mechanosensitive adapter protein Zyxin is a key example, integrating actin cytoskeleton dynamics with gene expression. However, the developmental regulation of its interactions and post-translational modifications remains poorly understood. Here, we characterize the dynamic Zyxin interactome across three early developmental stages of *Xenopus laevis* (from gastrulation to neurulation) using co-immunoprecipitation coupled with quantitative mass spectrometry (DDA and DIA). We identify stage-specific changes in Zyxin’s association with core focal adhesion components, transcriptional regulators and kinases. Furthermore, we uncover developmentally regulated phosphorylation events on isoforms, suggesting dynamic post-translational control of its interactions. Our work provides a comprehensive resource that positions Zyxin as a central orchestrator of cell adhesion, survival, and gene regulatory programs during morphogenesis. These findings underscore the role of Zyxin as a multifaceted regulatory hub, with important implications for understanding tissue homeostasis and related pathologies.

## 1. Introduction

Protein complexes function as dynamic molecular machines that govern cellular processes and organismal development [1]. Understanding their assembly mechanisms is therefore fundamental not only for deciphering normal embryogenesis but also for developing therapeutic strategies for developmental disorders [1,2]. The high conservation of many core complexes across species validates the use of model organisms, such as *Xenopus laevis*, to study principles of complex assembly relevant to human biology [3].

In this study, we use *Xenopus* embryos to investigate the dynamics of *zyxin*-associated protein assemblies during development by mass spectrometry. Zyxin is a LIM-domain protein with dual roles in the cytoplasm and nucleus. At focal adhesions, it regulates actin cytoskeleton assembly and turnover, thereby modulating cell migration, adhesion, and mechanotransduction [4]. Under specific conditions, Zyxin undergoes nuclear translocation, where it can act as a scaffold linking cytoskeletal dynamics to gene transcription, positioning it as a key integrator of morphogenetic movements and transcriptional responses [5,6].

This functional versatility is encoded within Zyxin’s modular structure. Its N-terminal domain mediates localization to adhesion sites via interactions with adhesion complexes components and is crucial for cytoskeletal remodeling [7]. A central nuclear export signal (NES) facilitates CRM1/Exportin-1-dependent nucleocytoplasmic shuttling [8,9]. The C-terminus contains three LIM domains that serve as a major protein-binding platform, enabling Zyxin to act as a scaffold by simultaneously engaging multiple partners [10,11]. Databases such as IntAct list over 100 potential interactors for human Zyxin (Supplementary Figure S1), underscoring its central role in interaction networks.

Critically, Zyxin’s interactions are regulated by post-translational modifications. Phosphorylation, particularly at residues such as Ser142 in mammals, can alter intramolecular interactions, expose binding sites, and thereby modulate its scaffold activity [12]. Despite this understanding, a comprehensive quantitative profile of the Zyxin interactome and its phospho-regulation during in vivo vertebrate development is still lacking.

To address this gap, we combined co-immunoprecipitation (co-IP) with advanced mass spectrometry to characterize the Zyxin-centered protein complex (interactome) and associated phosphorylation sites across three key early developmental stages in *Xenopus laevis* (from gastrulation to neurulation). Co-IP/MS is a powerful method for isolating a target protein together with its direct and indirect binding partners, providing insights into complex composition and dynamics. Using stage-specific embryos, our previously validated anti-Zyxin antibodies [13], and complementary high-resolution mass spectrometry approaches—Data-Dependent Acquisition (DDA) for deep identification and Data-Independent Acquisition (DIA) for robust quantification—we provide a comprehensive analysis of the dynamic Zyxin interactome during early vertebrate morphogenesis.

## 2. Results

### 2.1. Strategy for Mapping the Dynamic Zyxin Interactome During Early Development

**Figure 1:**
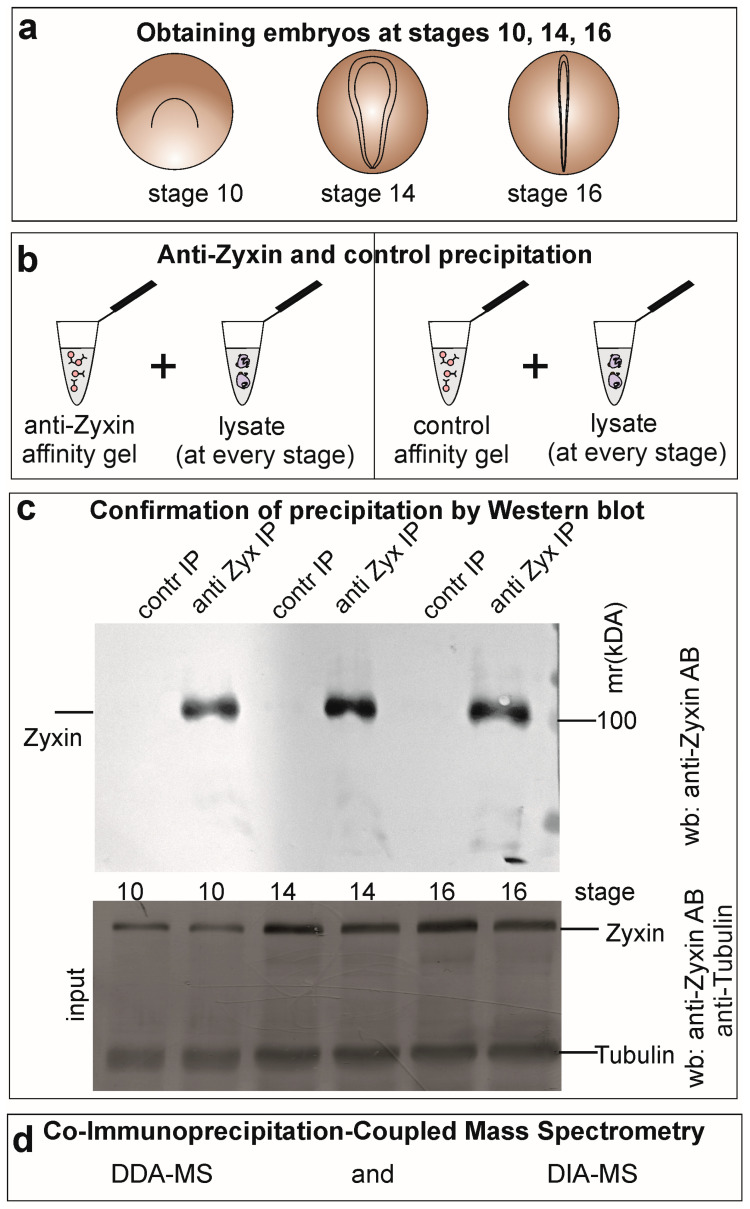
Stage-resolved analysis of the Zyxin interactome in *Xenopus laevis* embryos. (**a**) Schematic of the three analyzed developmental stages: early gastrula (stage 10), early neurula (stage 14), and late neural fold (stage 16). (**b**) Experimental workflow. Endogenous Zyxin complexes were isolated by co-immunoprecipitation (co-IP) from embryo lysates using a validated antibody cocktail and analyzed by quantitative mass spectrometry. (**c**) Western blot validation of co-IP specificity. Zyxin was efficiently and specifically immunoprecipitated across all stages. Non-specific rabbit IgG served as a negative control. Lysate inputs are shown as a reference. (**d**) Complementary mass spectrometry strategies: Data-Dependent Acquisition (DDA) for deep interactome discovery and Data-Independent Acquisition (DIA) for accurate, label-free quantification across stages.

To define stage-specific changes in the Zyxin protein interaction network (interactome), we performed co-immunoprecipitation (Co-IP) from lysates of *Xenopus laevis* embryos at three key developmental stages: early gastrula (stage 10), early neurula (stage 14), and late neural fold (stage 16) (Figure 1a). We employed a validated antibody cocktail targeting both the N- and C-terminal regions of Zyxin to maximize capture of native complexes [13], with non-specific rabbit IgG as a stage-matched negative control. Western blot analysis confirmed the efficient and specific immunoprecipitation of Zyxin at comparable levels across all stages (Figure 1b,c).

Immunoprecipitated complexes were analyzed by liquid chromatography-tandem mass spectrometry (LC-MS/MS). To ensure comprehensive and quantitative profiling, we used two complementary acquisition strategies: Data-Dependent Acquisition (DDA) for deep interactome discovery and Data-Independent Acquisition (DIA) for accurate label-free quantification across developmental stages (Figure 1d) [14,15,16]. Two independent biological replicates per stage were processed using both methods.

As Zyxin is a core component of large macromolecular assemblies like the integrin adhesome, our co-IP/MS approach captures both direct and indirect interactors. Therefore, to ensure biological relevance, we focused our subsequent analysis on proteins with established connections to Zyxin function, compiling a curated list from prior studies and major interaction databases (e.g., STRING, IntAct, GeneMANIA [17,18,19,20,21]). While novel candidates were identified, they require further validation and are not discussed here. This focused strategy enables us to report robust, stage-specific dynamics within the core Zyxin interaction network during early vertebrate morphogenesis.

### 2.2. The Zyxin-Centered Adhesome Core Is Stable, While Its Peripheral Interactions Are Developmentally Dynamic

**Figure 2:**
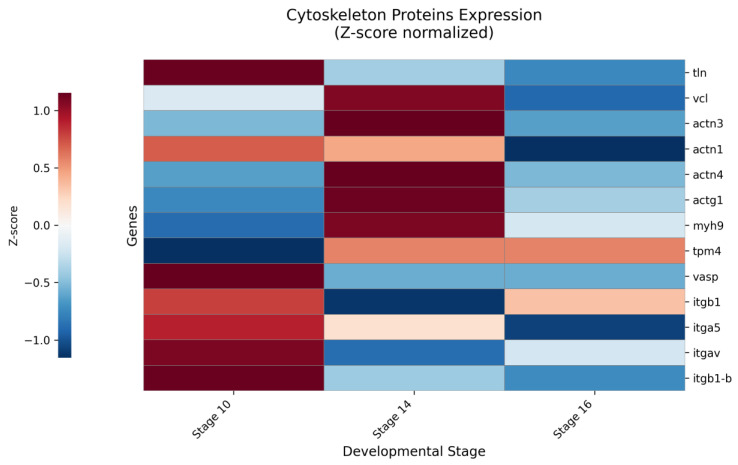
Relative stage-specific dynamics of Zyxin-associated structural and adhesion proteins. Heatmap depicts the stage-resolved association patterns of core cytoskeletal and focal adhesion proteins with the Zyxin complex. The color scale (red/blue) represents row-wise Z-scores, indicating whether the Z/C ratio for a given protein at a specific stage is above (red) or below (blue) its own mean value across all three stages. This normalization highlights the temporal dynamics of each protein relative to its own baseline. Notable patterns include the stable, constitutive association of the adhesome core components Talin-1 and Vinculin, the sharp, transient recruitment peak of α-actinin-3 and α-actinin-4 at stage 14, the progressive engagement of Myosin-9, and the absence of specific enrichment for Integrin subunits.

To elucidate how Zyxin-associated complexes are remodeled during morphogenesis, we performed a stage-resolved analysis of proteins co-precipitated with Zyxin, focusing on components of integrin-based adhesions and the actin cytoskeleton [22]. Quantitative MS analysis revealed a conserved, high-affinity core complex. Canonical focal adhesion (FA) linker proteins talin (*tln1*, *tln2*) and vinculin (*vcl*) were specifically enriched across all stages, confirming their role as stable, direct adapters within the Zyxin interactome (Figure 2, Table S1). In stark contrast, key Integrin subunits (*itga5*, *itgb1*) were detected but showed no specific enrichment compared to IgG controls (Z/CTRL < 1) at any stage (Figure 2, Table S1). This result directly validates the established adhesome architecture, wherein Zyxin’s connection to integrins is indirect and mediated by the stable Talin-Vinculin mechanical linkage [22] (Supplementary Figure S2).

The Actin Interface: A Dynamic Recruitment Hub. Consistent with Zyxin’s primary role in cytoskeletal organization [23,24,25], we observed robust co-precipitation of central actin-binding proteins. Cytoplasmic β-actin (*actb*) and cross-linking α-actinins (*actn1*, *actn4*) formed a stable, constitutive interface (Table S1). A comprehensive heatmap of quantitative enrichment across stages revealed that the recruitment of major cytoskeletal regulators into the Zyxin complex is developmentally regulated (Figure 2).

Notably, the specific enrichment of many core interactors was minimal at the onset of gastrulation (stage 10). This pattern shifted dramatically during neurulation (stages 14–16). We observed a significant, stage-specific amplification in the co-precipitation of key regulators, including α-actinin isoforms, Talin, and non-muscle Myosin II heavy chains (*myh9*, *myo10*) (Figure 2, Table S1). The canonical Zyxin effector VASP [26] (*vasp*) was among the most consistently and specifically enriched partners across all stages.

Collectively, these data delineate a bipartite architecture of the Zyxin interactome: an invariant mechanical core (Talin, Vinculin, Actin) ensures stable integration into the adhesion-cytoskeleton network. In parallel, developmentally modulated recruitment of regulators of Actin cross-linking (α-Actinins) and actomyosin contractility (Myosin II) peaks during neurulation. This stage-specific amplification of the interactome aligns with the intense mechanical demand and cytoskeletal remodeling required for neural tube formation, positioning Zyxin as a nucleating scaffold for force-responsive complexes during active morphogenesis.

The quantitative heatmap analysis revealed the dynamic recruitment of cytoskeletal proteins into the zyxin-associated complex across development (Figure 2). Notably, the specific enrichment of core interactors was minimal at the onset of gastrulation (stage 10). This pattern shifted dramatically during neurulation (stages 14–16), a period of intense morphogenetic movements, such as neural plate folding and convergent extension, which generate substantial morphogenetic tension. At these stages, we observed a significant increase in the co-precipitation of major cytoskeletal regulators, including α-actinin isoforms, Talin and non-muscle Myosin. This stage-specific amplification of the interactome correlates with the peak of mechanical forces and cytoskeletal remodeling essential for neural tube closure, suggesting that Zyxin acts as a nucleating scaffold for force-responsive complexes during active morphogenesis.

### 2.3. Stage-Specific Interactions with DNA-Binding Transcription Factors

Zyxin shuttles to the nucleus and can act as a transcriptional scaffold. Previously, yeast two-hybrid screens identified interactions between the Zyxin LIM domains and key developmental transcription factors, including the Sonic hedgehog (Shh) effectors Gli1 and Zic1, the repressor Xanf1, and Y-box binding proteins (YBX) [27,28,29,30]. These interactions were rigorously validated in vivo and in vitro using co-immunoprecipitation and pull-down assays with tagged proteins, confirming their biological relevance [27,28,29]. Here, using endogenous co-IP/MS, we assessed the developmental dynamics of these interactions in the native context of the embryo. We were unable to detect co-precipitation of the transcription factor Xanf1 with Zyxin by either DDA or DIA methods. This likely reflects the general technical challenge of detecting low-abundance transcription factors via mass spectrometry. In contrast, for other regulators such as YBX proteins and Gli1, we successfully identified specific, characteristic peptides using a combination of deep-coverage DDA sequencing and quantitative DIA, allowing us to confirm their stage-specific presence in the complex.

DDA mass spectrometric analysis revealed a stage-specific interaction with YBX family members. At the early gastrula stage (10), Zyxin co-precipitated with proteins identified by both conserved peptides (NGYGFINR, EDVFVHQTAIKK) shared by Ybx1 and Ybx2, as well as peptides unique to Ybx2 (GAEAANVTGPGGVPVK, VLATQVQGCTVK). By the early neurula stage (14), only the conserved signature peptide NGYGFINR was detected.

Our quantitative DIA analysis definitively identified the specific association of Ybx1, Ybx2 factors with Zyxin, detecting 10 unique peptides. The most specific signal for this interaction was observed at developmental stage 14, exemplified by peptides AGQEPAATVGEK (Ybx1) and NDTKEDVFVHQTAIK (Ybx2). This finding is particularly significant as proteins like Ybx1, Ybx2 known for their promiscuous RNA-binding and potential cytoskeletal associations, typically generate high non-specific background in co-IP assays, obscuring specific interactions. The ability of our DIA workflow to clearly resolve this stage-specific signal above the expected noise validates its sensitivity and precision in mapping dynamic protein complexes under challenging conditions.

For the transcription factor Gli1, a previously confirmed partner [28], we observed a distinct temporal pattern. Gli1 was not detected in Zyxin complexes at stages 10 or 14. However, at stage 16, a specific Gli1 peptide (LGLLDGR, residues 226–232) was identified in the DDA dataset (Table S2). This peptide was not captured in the parallel DIA analysis, which can be attributed to its small size (7 aa) placing it below the optimal *m*/*z* range for our DIA method settings. This result suggests a late-stage specific association between Zyxin and Gli1, consistent with the peak activity of Hedgehog signaling during neural patterning. The complementary use of both MS methods was thus crucial for identifying this low-abundance interaction.

Our endogenous co-IP/MS analysis maps the developmental timing of Zyxin’s association with specific transcription factors, revealing highly dynamic, stage-restricted interactions. The previously established link to Y-box proteins [29] is transient, peaking during gastrulation and early neurulation before ceasing by stage 16. Conversely, the biochemically confirmed interaction with the Sonic hedgehog effector Gli1 [28] emerges specifically at the late neural fold stage, coinciding with the peak of Hedgehog signaling during neural patterning. These distinct temporal profiles demonstrate that Zyxin acts as a developmentally programmed scaffold, assembling specific transcriptional complexes in phase with morphogenetic transitions. The early window of Ybx interaction may link Zyxin to mRNA regulation during rapid cell movements, while its late-stage recruitment of Gli1 could modulate Hedgehog-dependent transcription during neural tube formation.

Collectively, these findings transform our understanding of Zyxin from a static cytoskeletal component to a dynamic nuclear integrator. Its ability to sequentially recruit distinct, validated transcriptional regulators (Ybx1, Ybx2, Gli1) positions Zyxin as a key node in coordinating stage-specific gene expression programs with the mechanical and signaling demands of embryogenesis.

### 2.4. Developmental Regulation of Zyxin Isoforms and Phosphorylation

Phosphorylation is a key regulator of Zyxin’s scaffold function. To map its phosphorylation dynamics during early *Xenopus laevis* development, we employed a complementary mass spectrometry strategy. While Data-Dependent Acquisition (DDA) is well-suited for the initial discovery of high-abundance phosphopeptides, its sensitivity for detecting low-stoichiometry phosphorylation events is limited. Therefore, we complemented DDA with Data-Independent Acquisition (DIA), which provides superior quantitative accuracy and depth for profiling dynamic post-translational modifications.

Stage- and Isoform-Specific Phosphorylation in the N-terminal Regulatory Domain.

Initial DDA analysis identified two distinct phosphopeptides in the N-terminal region homologous to the regulatory human Ser142 (Table 1 and Table S3) [12,31,32,33]. Crucially, these peptides originate from different Zyxin isoforms with alternative start codons, leading to distinct amino acid numbering. Aligning the sequences to the full-length protein structure reveals that the phosphorylated serine in both peptides corresponds to the same conserved structural position within the N-terminal regulatory domain:

- Peptide “3P” (SSPPPAFPKPEPPSVAPK, isoform A5H447): Phosphorylated at Ser198 (numbered within this isoform).
- Peptide “4P” (SPPPPAFPKPEPPSVAPK, isoforms A0A8J1LC30/A0A974H9B9): Phosphorylated at Ser250 (numbered within these isoforms), which is structurally equivalent to Ser198 in A5H447.

Quantitative DIA profiling precisely resolved the developmental dynamics of both N-terminal phosphoforms, revealing distinct, isoform-specific regulation. For the phosphopeptide from isoforms A0A8J1LC30/A0A974H9B9 (“4P” peptide, pSer250), the stoichiometry of phosphorylation decreased sharply between gastrulation and neurulation—the phospho/non-phospho ratio fell from 0.26 at stage 10 to 0.09 at stage 14 (Table 1 and Table S3), indicating a pronounced down-regulation within this isoform group during early morphogenesis. Analysis of the phosphopeptide from isoform A5H447 (“3P” peptide, pSer198) revealed a distinct temporal pattern: it was fully phosphorylated at stage 10, but its phospho/non-phospho ratio exhibited a clear decline at later stages, dropping from 1.2 at stage 14 to 0.8 at stage 16 (Table 1). Therefore, although phosphorylation of the conserved N-terminal site is progressively down-regulated in both major isoforms, the timing is distinct—the “4P” form declines early (gastrula to neurula), whereas the “3P” form, starting from a fully modified state, declines later (neurula to late neural fold).

DIA Uncovers a Complex Phosphorylation Landscape Beyond DDA Detection.

The enhanced sensitivity of DIA was crucial for mapping phosphorylation outside the N-terminus (Table 1 and Table S3). Analysis of the central domain peptide SLGPQTESG**RSPGAQ**STGGK revealed constitutive phosphorylation at Ser376 and Ser383 across all stages (relative ratios: 0.06–0.14), and developmentally regulated phosphorylation at the conserved Ser386, present at stages 10 and 14 but absent at stage 16.

A peptide from the central region of the Zyxin molecule, SLGPQTESG**RSPGA**QSTGGK (residues 376–395), phosphorylated at Ser386, is present in all Zyxin isoforms. The sequence **RSPGA** (highlighted in bold), containing Ser386, is identical to the corresponding region in HEK293T, HeLa, RKO, and SW480 human cell lines [34] (Figure S3). The high conservation of these phosphorylation sites suggests that this modification occurs during mitosis, as in human cell lines [34]. We analyzed the degree of phosphorylation of this peptide at selected developmental stages and found it to be very low, and it was not detected at all at the neurula stage (Table 1). The low abundance of the phosphorylated form can be explained by its appearance only in dividing cells during mitotic metaphase.

Most notably, DIA uniquely identified low-abundance dual phosphorylation within the first LIM domain (peptide AGEHLYHVACFTCSR, pThr499/pSer501). The estimated stoichiometry was exceptionally low (ratios~10^−5^), explaining why this novel regulatory site was not captured by the less sensitive DDA method.

Functional Validation of N-terminal Phosphorylation in Nuclear Trafficking.

To probe the functional role of the identified dynamic phosphorylation sites, we performed a preliminary structure-function analysis. We generated phospho-deficient (S198A, S386A) and phospho-mimetic (S198D, S386D) mutants of *Xenopus* Zyxin at two key sites: the stage-specifically regulated N-terminal Ser198 and the conserved central Ser386.

Expression of these mutants in embryos did not induce overt morphological defects, likely due to the presence of a substantial maternal pool of wild-type Zyxin, which complicates loss-of-function analysis. However, cellular fractionation experiments revealed a specific role for the N-terminal site. While mutation of Ser386 did not alter Zyxin’s nucleo-cytoplasmic distribution, both the S198A and S198D mutations significantly impaired its nuclear accumulation (Figure S4). This result indicates that the phosphorylatable state of Ser198, rather than a simple charge mimicry, is critical for efficient nuclear import.

This finding aligns with the established molecular mechanism in mammalian cells, where phosphorylation at the homologous site (Ser142) creates a docking site for 14-3-3 proteins, which facilitate nuclear translocation [33]. In summary, our initial functional data directly link the dynamic phosphorylation at Ser198—which we observed to peak during gastrulation—to the regulation of Zyxin’s subcellular localization.

Our phosphoproteomic analysis reveals that Zyxin is subject to sophisticated, multi-layered regulation during development. Phosphorylation is domain-specific, isoform-specific, and stage-specific. The N-terminal regulatory site is most active early, while the central region gains a distinct modification later. The discovery of phosphorylation within the LIM domain itself suggests a novel mechanism for directly modulating Zyxin’s scaffold function. These dynamic patterns position phosphorylation as a central mechanism tuning Zyxin’s role as a mechanosensitive hub across different morphogenetic events.

### 2.5. Zyxin Interacts with Kinases, Apoptotic Regulators, and Signal Transducers

To elucidate potential mechanisms regulating Zyxin’s phosphorylation and function, we analyzed kinases and regulatory proteins co-precipitating with Zyxin across developmental stages. We focused our analysis on a curated set of proteins with well-documented, direct interactions with Zyxin, as established in the literature (14-3-3, Akt, CDK, Caspase [33,34]). This strategy allowed us to filter the complex proteomic data for biologically relevant changes within a high-confidence interactome.

Our data align with and extend established phosphorylation pathways. In human cells, AKT kinase phosphorylates Zyxin at Ser142, creating a binding site for 14-3-3 proteins. This interaction promotes Zyxin’s nuclear translocation and complex formation with Acinus, suppressing apoptosis [33]. In our *Xenopus* study, AKT2 isoforms (AKT2A/B) were specifically detected in Zyxin complexes, most prominently at stage 10. This temporal association coincides with a high phosphorylation signal on the homologous site, suggesting a conserved role for AKT in regulating Zyxin during early morphogenesis. Furthermore, multiple 14-3-3 protein isoforms (YWHAB, YWHAG, YWHAZ, YWHAE) were robustly and specifically enriched in our interactome, strongly corroborating the functional link between Zyxin phosphorylation and 14-3-3 binding in vivo.

**Figure 3:**
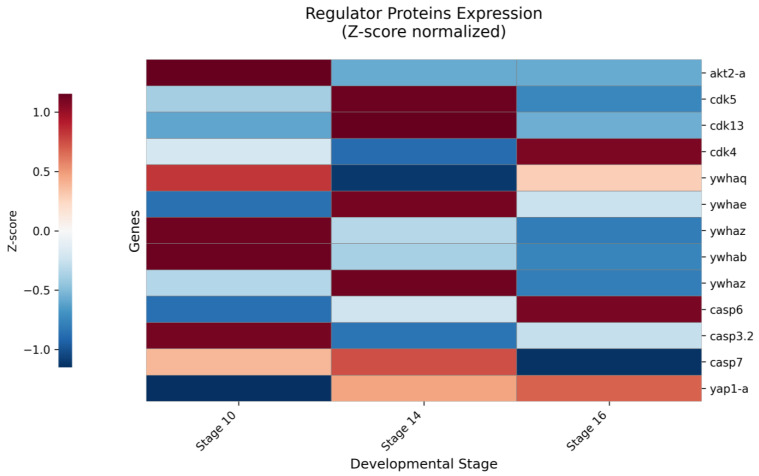
Relative stage-specific dynamics of Zyxin-associated regulators. Heatmap depicts the stage-resolved association patterns of regulatory proteins with the Zyxin complex. The color scale (red/blue) represents row-wise Z-scores, indicating whether the Z/C ratio for a given protein at a specific stage is above (red) or below (blue) its own mean value across all three stages. This normalization highlights the temporal dynamics of each protein relative to its own baseline. Notable patterns include the constitutive, high-level signal of Caspase-3 (*casp3.2*), the late-stage-specific emergence of YAP1-A, and the transient peak of CDK4 at stage 16.

Another critical regulatory axis involves cell-cycle kinases. Zhou et al. showed that Cyclin-Dependent Kinase 1 (CDK1) phosphorylates Zyxin during mitosis to promote cancer cell proliferation, and linked Zyxin to the transcriptional co-activator YAP via CDK8 [34]. Analysis of cyclin-dependent kinase 1 (CDK1) revealed a nuanced association with the Zyxin complex. While both CDK1-A and CDK1-B isoforms were detectable by deep-coverage DDA sequencing at stage 16, they showed no quantitative enrichment in our comparative DIA measurements across development. This suggests that CDK1 may be present within the broad Zyxin-associated protein network but does not form a stable, stoichiometric complex under the conditions captured by our assay (Figure 3).

Quantitative analysis of other cyclin-dependent kinases (CDKs) revealed their stage-specific association with the Zyxin complex. CDK5 and CDK13 showed consistent, moderate enrichment across developmental stages. In contrast, the signal for CDK4 was highly variable, with a notable enrichment specifically at stage 16, suggesting a potential late-stage regulatory role.

While CDK8 was not found, the downstream effector Yap1 was specifically co-precipitated at stages 14 and 16. This suggests a developmentally regulated Zyxin-YAP interaction, potentially independent of CDK8, meriting further investigation [35].

We also identified caspases within the Zyxin complex. Chan et al. demonstrated that human Zyxin is a substrate for Caspase-3 in vitro [33]. Our study provides in vivo evidence, as Caspase-3.2 (*casp3.2*) co-precipitated with endogenous Zyxin with high specificity across stages, with lower levels of Caspase-6, -7, and -10 also detected. This finding strongly supports a physiological role for caspase-mediated cleavage in Zyxin turnover during development (Figure 3, Table S4).

The quantitative Zyxin interactome maps its position within a multifaceted signaling network. Stage-specific associations with AKT and CDKs correlate with its phosphorylation dynamics. The relationship between Zyxin and CDKs appears multifaceted. This highlights that Zyxin’s regulation by these kinases may involve brief, catalytic encounters distinct from the stable scaffolding interactions that define its core complex. The functional outcome of this CDK-mediated phosphorylation on Zyxin’s activity during morphogenesis warrants further investigation. Stable, high-affinity interactions with 14-3-3 proteins and Caspases confirm conserved roles in survival and apoptosis. Finally, the late-emerging link to YAP highlights a potential pathway through which Zyxin could transduce mechanical or adhesive cues into transcriptional outputs during embryogenesis.

## 3. Discussion

Our stepwise quantitative proteomic analysis provides the first dynamic map of the protein complex containing the mechanosensitive scaffold Zyxin during early vertebrate morphogenesis. The data reveal that this complex is not a static entity but undergoes precise compositional and post-translational remodeling. This dynamic behavior is governed by a two-component architectural principle: an evolutionarily conserved structural core and a dynamically recruited stage-specific regulatory periphery.

### 3.1. A Conserved Core and a Developmentally Dynamic Periphery

A central finding is the identification of an invariant structural core, defined by the consistent, specific co-immunoprecipitation of the essential focal adhesion adaptors Talin and Vinculin with Zyxin across all stages. This observation firmly anchors Zyxin within the canonical integrin adhesome via an indirect linkage, corroborating biochemical models from mammalian cell studies in an embryonic context [36,37,38]. The weak specific enrichment of integrins themselves aligns with this established model.

In stark contrast, the regulatory periphery of the complex exhibits striking stage-specific dynamics. The pronounced, transient recruitment of α-Actinin isoforms during early neurulation signals a period of enhanced Actin crosslinking and cytoskeletal reinforcement, a prerequisite for tissue folding. Concurrently, non-muscle Myosin II (*myh9*) displays progressively increasing association, indicating quantitative, stage-dependent tuning of actomyosin contractility. This dynamic periphery allows the complex to function as a tunable hub, meeting stage-specific mechanical demands. Heatmap analysis visually summarizes this functional transition: between gastrula and neurula stages, Zyxin-associated complexes shift from an integrin-adhesion-centric to an actomyosin-centric composition, correlating with the transition from intensive cell migrations to the formation of stable, actin-bundle-rich structures [39,40,41,42,43,44] (Figure 2).

### 3.2. A Multi-Layered Phosphoregulatory Code with Functional Output

Our integrated analysis reveals that Zyxin is regulated by a sophisticated, developmentally timed phosphorylation code, that is dynamically timed to developmental stages. By employing complementary DDA and DIA mass spectrometry, we mapped dynamics that were previously inaccessible. We identified isoform-specific regulation at the conserved N-terminal regulatory site [12,31,33]. This indicates that different Zyxin isoforms are subject to distinct temporal control. Mutational analysis demonstrated that the phosphorylatable state of Ser198 is essential for efficient nuclear import, providing a direct molecular explanation for how stage-specific phosphorylation could regulate Zyxin’s access to its nuclear interaction partners. Beyond the N-terminus, DIA uncovered constitutive phosphorylation at Ser376/Ser383, stage-regulated phosphorylation at Ser386 (absent by stage 16), and a novel, low-abundance phosphorylation within the first LIM domain (Thr499/Ser501), suggesting a new layer of regulatory potential. Collectively, these dynamic patterns position phosphorylation as a central switchboard that likely reconfigures the Zyxin interactome to meet the changing mechanical and signaling demands of gastrulation and neurulation.

### 3.3. Zyxin as an Integrative Node for Signaling and Transcriptional Regulation

Beyond its role in cytoskeletal remodeling, our interactome data position Zyxin as a central node within broader signaling networks. The robust co-immunoprecipitation of 14-3-3 proteins and Caspases provides strong in vivo evidence for Zyxin’s physical connection to apoptosis regulation. Associations with kinases like AKT2 place the complex at the intersection of survival pathways.

Furthermore, we demonstrate stage-specific interactions between Zyxin and DNA-binding transcription factors. The transient association with Y-box proteins (Ybx1) during gastrulation correlates with the maternal-to-zygotic transition, while the specific co-precipitation of Gli1 at the late neurula stage coincides with the peak of Hedgehog signaling during neural patterning [28,29]. These data integrate the cytoskeletal hub with key developmental pathways, supporting the hypothesis that Zyxin acts as a temporal scaffold, assembling distinct transcriptional modules to coordinate gene expression with morphogenetic progression.

### 3.4. Conclusions and Implications

In summary, our integrated analysis redefines Zyxin from a static structural component to a developmentally reprogrammable signaling hub. Its interactome is architecturally organized into a stable core for mechanical integration and a dynamic periphery for regulatory input, with its function further tuned by a multi-layered phosphoregulatory code. By serving as a dynamic interface that connects adhesion sites to the cytoskeleton, apoptotic machinery, and stage-specific transcriptional regulators, Zyxin emerges as a central integrator tasked with coordinating mechanical and biochemical cues essential for vertebrate morphogenesis. The evolutionary conservation of its key interactions underscores their fundamental importance and suggests that dysregulation of this dynamic assembly process may contribute to disease states characterized by aberrant mechanosensing [36]. This resource provides a molecular roadmap for future investigations into the logic of scaffold protein regulation in development and disease.

## 4. Materials and Methods

The following reagents, antibodies, software, and instrumentation were used in this study. Their details, including manufacturers, catalog numbers, and software versions, are summarized in Table 2 (Key Resources Table).

### 4.1. Embryo Manipulations

All animal care and handling were conducted in accordance with the regulations of the Animal Committee at the Shemyakin-Ovchinnikov Institute of Bioorganic Chemistry (Moscow, Russia), the Animals (Scientific Procedures) Act of 1986, and the Helsinki Declaration.

*Xenopus laevis* embryos were obtained by in vitro fertilization [45]. Mature female frogs were hormonally induced to ovulate by injection of human chorionic gonadotropin (hCG). Eggs were collected and fertilized with sperm obtained from dissected testes of male frogs. The sperm suspension was added to the eggs in 1× Marc’s Modified Ringer’s (MMR) solution to facilitate fertilization. After fertilization, embryos were dejellied by brief incubation in a 2% cysteine solution (pH 7.8) to remove the jelly coat. Following several washes in 0.1× MMR to remove cysteine, embryos were cultured at 18–20 °C in 0.1× MMR solution throughout early development [45].

Embryos were staged according to the Nieuwkoop and Faber standard table [46].

### 4.2. Zyxin Co-Immunoprecipitation from Embryo Lysates

#### 4.2.1. Preparation of Antibody-Conjugated Resin

Protein A Sepharose beads (Sigma) were washed three times with PBS and then blocked with 5% Bovine Serum Albumin (BSA) in PBS for 1 h at 4 °C on a rotator. The beads were then divided into aliquots. For the specific immunoprecipitation, an aliquot of beads was incubated with 30 µL of polyclonal rabbit anti-C-Zyxin antibodies and 20 µL of polyclonal rabbit anti-N-Zyxin antibodies (previously characterized in [13]) for 3 h at 4 °C on a rotator. For the control precipitation, an equivalent aliquot of beads was incubated with the same volume (50 µL) of non-immune rabbit IgG (Sigma). Following incubation, the beads were washed three times with PBS to remove unbound antibodies.

#### 4.2.2. Embryo Lysis and Sample Preparation

For each biological replicate, a pool of 100 embryos at the desired developmental stage (stages 10, 14, or 16) was homogenized on ice in 200 µL of Immunoprecipitation Buffer (IPB: 20 mM HEPES pH 7.6, 2% sucrose, 150 mM KCl, 1.5 mM MgCl_2_, 0.2 mM EDTA, 0.5 mM DTT, 1% NP-40) supplemented with protease (Sigma-Aldrich, St. Louis, MO, USA, P8340, 1:50 dilution) and phosphatase (Sigma-Aldrich, St. Louis, MO, USA, P0044, 1:100 dilution) inhibitor cocktails. The homogenate was centrifuged at 16,000× *g* for 30 min at 4 °C to pellet yolk platelets and cellular debris. The clarified supernatant (lysate) was collected, and its total protein concentration was determined using a Bradford assay (Bio-Rad). The approximate total protein yield obtained from 100 embryos was 1 mg for stages 10 and 14, and 1–1.2 mg for stage 16.

#### 4.2.3. Immunoprecipitation Procedure

Equal amounts of total protein from each lysate (approximately 500 µg) were incubated overnight at 4 °C on a rotator with the prepared aliquots of antibody-conjugated beads (anti-Zyxin or control IgG). Following incubation, the beads were washed three times with 1 mL of cold Wash Buffer (PBS).

Following the final wash, the beads with bound immunocomplexes were divided for parallel analysis.

For Western blot analysis, proteins were eluted from a portion of the beads by incubation with 40 µL of 2× Laemmli sample buffer containing 5% β-mercaptoethanol at 95 °C for 5 min. An aliquot of this eluate, equivalent to material from 10 embryos, was used for SDS-PAGE and immunoblotting.

For mass spectrometric analysis, the remainder of the beads with the bound protein complexes was processed directly via on-bead trypsinolysis, as described in Section 4.5.1.

### 4.3. Nuclear-Cytoplasmic Fractionation

Nuclear and cytoplasmic fractions were isolated from *Xenopus laevis* embryos using a digitonin-based permeabilization protocol adapted from Martynova et al. [45]. Briefly, staged embryos were treated with 150 µg/mL cycloheximide in 0.1× MMR for 1 h to arrest translation. For each sample, approximately 30 embryos were lysed in ice-cold Isotonic Lysis Buffer (20 mM HEPES pH 7.4, 2% sucrose, 10 mM KCl, 1.5 mM MgCl_2_, 0.2 mM EDTA, 0.5 mM DTT, protease inhibitors) containing 0.05% digitonin. The lysate was centrifuged at 5000× *g* for 8 min at 4 °C to pellet the nuclei. The supernatant (cytoplasmic fraction) was collected and clarified by further centrifugation. The nuclear pellet was washed and subsequently extracted with a high-salt buffer (Isotonic Lysis Buffer supplemented with 150 mM KCl and 0.5% NP-40) for 45 min at 4 °C. The soluble nuclear fraction was recovered by centrifugation at 14,000× *g* for 10 min. Proteins from both fractions were concentrated by methanol-chloroform precipitation, resolved by SDS-PAGE, and analyzed by Western blot. Fraction purity was validated using antibodies against histone H3.

### 4.4. SDS-PAGE and Western Blotting

Samples were separated by SDS-PAGE on 10% polyacrylamide gels according to the method of Laemmli. Proteins were then transferred onto a PVDF membrane (Millipore) using a semi-dry blotting system. The membrane was blocked with 5% non-fat milk in TBST (tris-buffered saline with 0.1% Tween-20) for 1 h at room temperature.

For immunodetection, the membrane was incubated overnight at 4 °C with primary anti-C-Zyxin antibodies [13] diluted 1:1000 in blocking buffer. After washing, the membrane was incubated for 1 h at room temperature with goat anti-rabbit IgG secondary antibodies conjugated to alkaline phosphatase (Sigma) diluted 1:5000. The signal was developed using the NBT/BCIP chromogenic substrate (Promega). To confirm equal loading, membranes were stained with Ponceau S solution after development or re-probed with antibodies against a housekeeping protein (e.g., β-actin). Band intensities were quantified using ImageJ software (National Institutes of Health).

### 4.5. Mass Spectrometric Analysis

#### 4.5.1. Sample Preparation for Mass Spectrometry

Proteins co-immunoprecipitated on Protein A Sepharose beads were processed for mass spectrometry using on-bead trypsinolysis with sodium deoxycholate (SDC). Reduction, alkylation and digestion of the proteins were performed as described previously [47] with minor modifications. Briefly, 20 µL of sodium deoxycholate (SDC) reduction and alkylation buffer pH 8.5 contained 100 mM TRIS, 1% (*w*/*v*) SDC, 10 mM TCEP and 20 mM 2-chloroacetamide were added to a protein sample. The sample was heated at 85 °C for 10 min, cooled to a room temperature and 0.1 γ trypsin in 5 µL of 100 mM TRIS pH 8.5 was added. After overnight digestion at 37 °C, peptides were acidified by 50 µL of 2% trifluoroacetic acid (TFA) mixed with 50 µL of ethyl acetate and loaded on SDB-RPS StageTips contained two 14-gauge SDB-RPS plugs, and the StageTip was centrifuged at 300× *g* until all solution go through the StageTip (typically 4 min). After washing the StageTips with a 100 µL of 1% TFA/ethyl acetate 1:1 mixture (2 times) and 50 µL of 0.2% TFA, peptides were eluted in a clean tube by 60 µL 60% acetonitrile/5% ammonia mixture using centrifugation at 300× *g*. The collected material was vacuum-dried and stored at −80 °C. Before analyses peptides were dissolved in 20 µL of 2% acetonitrile/0.1% TFA and sonicated for 1 min.

#### 4.5.2. Liquid Chromatography and Mass Spectrometry (DDA Analysis)

Samples were loaded to a home-made trap column 50 × 0.1 mm, packed with Reprosil-Pur 200 C18-AQ 5 μm (Dr. Maisch), in the loading buffer (2% ACN, 98% H_2_O, 0.1% TFA) at 4 μL/min flow and separated at RT in a home-packed [48] fused-silica column 300 × 0.1 mm packed with Reprosil-Pur C18-AQ 1.9 μm (Dr. Maisch) into an emitter prepared with P2000 Laser Puller (Sutter, USA). Reverse-phase chromatography was performed with an Ultimate 3000 Nano LC System (Thermo Fisher Scientific), which was coupled to the Orbitrap Tribrid Lumos mass spectrometer (Thermo Fisher Scientific) via a nanoelectrospray source (Thermo Fisher Scientific). Water containing 0.1% (*v*/*v*) FA was used as mobile phase A and ACN containing 0.1% FA (*v*/*v*), 20% (*v*/*v*) H_2_O as mobile phase B. Peptides were eluted from the trap column with a linear gradient: 3–5% B for 5 min, 5–35% B for 100 min, 35–60% B for 20 min, 60% B during 3 min, 60–99% B for 0.1 min, 99% B during 10 min, 99–2% B for 0.1 min at a flow rate of 500 nL/min. MS data was collected in DDA mode. MS1 parameters were as follows: 60 K resolution, 350–1600 scan range, max injection time—auto, AGC target—standard. Ions were isolated with 1.2 *m*/*z* window, preferred peptide match and isotope exclusion. Dynamic exclusion was set to 20 s. MS2 fragmentation was carried out in HCD mode at 15 K resolution with HCD collision energy 30%, max injection time—80 ms, AGC target—standard. Other settings: charge exclusion—unassigned, 1, >6.

#### 4.5.3. Mass Spectrometry Data Analysis (DDA Data)

MS raw files were analyzed by Peaks studio 11 (Bioinformatics Solutions Inc.) [49]. Identification of proteins was made by searching against the databases for *Xenopus laevis* (Uniprot version of 03.2025) with a carbamidomethyl Cys as a fixed modification, acetylation (N-term), deamidation Asn/Gln and Met oxidation as variable modifications. Additional PTMs were searched using all Peaks studio 11 built-in modifications. False discovery rate for peptide-spectrum matches was determined by searching a reverse database and was set to 0.01. Enzyme specificity was set as C-terminal to arginine and lysine (semi-specific), and a maximum of three missed cleavages were allowed in the database search. Peptide identification was performed with an allowed initial precursor mass deviation up to 10 ppm and an allowed fragment mass deviation 0.02 Da.

#### 4.5.4. DIA-LC-MS Analysis

DIA-LC-MS analysis was performed as described previously with minor modifications [50]. Peptides were loaded to a home-made trap column 50 × 0.1 mm, packed with Reprosil-Pur 200 C18-AQ 5 μm (Dr. Maisch), in the loading buffer (2% ACN, 98% H_2_O, 0.1% TFA) at 4 μL/min flow and separated at RT in a home-packed fused-silica column 300 × 0.1 mm packed with Reprosil-Pur C18-AQ 1.9 μm (Dr. Maisch) into an emitter prepared with P2000 Laser Puller (Sutter, USA). Reverse-phase chromatography was performed with an Ultimate 3000 Nano LC System (Thermo Fisher Scientific), which was coupled to the Orbitrap Tribrid Lumos mass spectrometer (Thermo Fisher Scientific) via a nanoelectrospray source (Thermo Fisher Scientific). Water containing 0.1% (*v*/*v*) FA was used as mobile phase A and ACN containing 0.1% FA (*v*/*v*), 20% (*v*/*v*) H_2_O as mobile phase B. Peptides were eluted from the trap column with a linear gradient: 3–6% B for 5 min, 6–35% B for 53 min, 35–60% B for 4 min, 60% B during 6 min, 60–99% B for 0.1 min, 99% B during 7 min, 99–2% B for 0.1 min at a flow rate of 500 nL/min. MS data was collected in DIA mode. In overlapping window DIA-MS parameters, MS1 spectra were collected in the range of *m*/*z* 495–745 at 15,000 resolution to set an AGC target—standard. MS2 spectra were collected at *m*/*z* 200–1800 at 50,000 resolution to set a normalized AGC target of 2000%, a maximum injection time of “auto”, and stepped normalized collision energies of 22, 26, and 30%. The width of the isolation window was set to 4 Da, and overlapping window patterns at *m*/*z* 500–740 were used for window placements (Table S5).

#### 4.5.5. DIA-NN Data Analysis

Search parameters of DIA-NN [51] (version 2.2.0) were set as follows: precursor FDR 1%; scan window set to 0; isotopologues and MBR turned on; protein inference at gene level; heuristic protein inference enabled; quantification strategy set to Quant UMS (high precision); neural network classifier single-pass mode (cross-validated); mass accuracy at MS1 and MS2 set to both 0. The settings for in silico library generation from a protein sequence database Xenopus laevis (25 March 2025) were as follows: Trypsin/P with maximum 1 missed cleavage; protein N-terminal M excision on; Carbamidomethyl on C as fixed modification; oxidation M and Ac (N-term) as variable modifications; maximum variable modifications 1; peptide length from 7 to 30; precursor charge 1–4; precursor *m*/*z* from 300 to 1800; fragment *m*/*z* from 200 to 1800.

### 4.6. Quantitative Analysis and Data Visualization

For data visualization, heatmap analysis was performed using Python 3.12.7 with pandas (v2.3.3), numpy (v2.3.4), and seaborn (v0.13.2) libraries. Expression data were Z-score normalized per protein to visualize relative changes across developmental stages. The ‘RdBu_r’ colormap was used where red/blue indicates expression above/below each protein’s mean. Figures were exported as 300 DPI TIFF files. Two primary heatmaps were constructed: (1) the expression dynamics of cytoskeleton proteins at stages 10, 14, and 16; (2) the co-immunoprecipitation profiles of Zyxin-interacting proteins.

Statistical significance of differences in protein expression and phosphorylation levels between stages was assessed using Student’s *t*-test with Benjamini–Hochberg correction for multiple testing (FDR < 0.05). Hierarchical clustering was performed using Euclidean distance with complete linkage. Functional enrichment analysis was conducted using Gene Ontology (GO) term enrichment and KEGG pathway analysis through the clusterProfiler package in R (version 4.10.0).

### 4.7. Experimental Design and Data Availability

The analysis of protein-protein interactions was focused on components of integrin-based adhesions and the actin cytoskeleton, as Zyxin is a core component of these structures. The conserved architecture of the integrin adhesome in vertebrates [52] (Supplementary Figure S2) validates the relevance of the selected targets for study in the Xenopus laevis model.

All co-immunoprecipitation experiments followed by mass spectrometry were performed in duplicate for each developmental stage (stages 10, 14, and 16) and for each acquisition method (DDA and DIA). The resulting datasets were integrated for subsequent bioinformatic analysis.

The mass spectrometry proteomics data have been deposited to the ProteomeXchange Consortium via the PRIDE partner repository with the dataset identifiers PXD071213 and PXD071283.

## Figures and Tables

**Table 1:** DIA Mass Spectrometric Analysis of Zyxin Phosphosite Dynamics.

Peptide (Phosphorylated Form)	Modification(s)	Zyxin Isoform (UniProt)	Residue Numbers for A5H447	Stage 10.5 Ratio (Phosph/Non-Phosph)	Stage 14 Ratio (Phosph/Non-Phosph)	Stage 16 Ratio (Phosph/Non-Phosph)
SSPPPAFPKPEPPSVAPK	pSer198	A5H447	197–214	(all detected signal)	1.2	0.8
SPPPPAFPKPEPPSVAPK	pSer197	A0A811LC30, A0A974HB89	197–214	0.26	0.05	0.03
SLGPQTESGRSPGAQSTGGK	pSer376	A5H447, A0A811LC30, A0A974HB89	376–395	High	High	High
SLGPQTESGRSPGAQSTGGK	pSer383	A5H447, A0A811LC30, A0A974HB89	376–395	High	High	High
SLGPQTESGRSPGAQSTGGK	pSer386	A5H447, A0A811LC30, A0A974HB89	376–395	High	High	0
AGEHLYHVACFTCSR	pThr499, pSer501	A5H447	488–502	0.00008	0.00004	0

DIA-based mass spectrometric analysis of Zyxin phosphorylation-site dynamics across embryonic stages. The table lists phosphopeptides, modified residues and isoforms, together with phospho/non-phospho signal ratios at stages 10.5, 14 and 16. Phosphorylated amino acid residues are underlined.

**Table 2:** Key Resources Table.

Reagent or Resource	Source	Identifier
ANTIBODIES		
Rabbit polyclonal anti-C-Zyxin	A.G. Zaraisky lab, Moscow, Russia	N/A
Rabbit polyclonal anti-N-Zyxin	A.G. Zaraisky lab, Moscow, Russia	N/A
Rabbit monoclonal anti-Histone H3	Abcam, Cambridge, UK	Cat# ab1791; RRID:AB_302613
Anti-rabbit IgG, AP-conjugated, produced in goat	Sigma-Aldrich, St. Louis, MO, USA	Cat#A3937; RRID:AB_258122
CHEMICALS, PEPTIDES, AND RECOMBINANT PROTEINS		
Protein A-sepharose	Sigma-Aldrich, St. Louis, MO, USA	Cat#P3391
Human chorionic gonadotropin (hCG)	Sigma-Aldrich, St. Louis, MO, USA	Cat#CG10
Western Blue^®^ stabilized substrate for alkaline phosphatase	Promega, Madison, WI, USA	Cat#S3841
Igepal Nonidet P-40	Sigma-Aldrich, St. Louis, MO, USA	Cat#I3021
DTT	Thermo Fisher Scientific, Waltham, MA, USA	Cat#R0861
Sodium deoxycholate (SDC)	Sigma-Aldrich, St. Louis, MO, USA	Cat#D6750
Trifluoroacetic acid (TFA), MS grade	Sigma-Aldrich, St. Louis, MO, USA	Cat#T6508
2-Chloroacetamide (CAA)	Sigma-Aldrich, St. Louis, MO, USA	Cat#22790
Tris(2-carboxyethyl)phosphine (TCEP)	Sigma-Aldrich, St. Louis, MO, USA	Cat#C4706
Trypsin, sequencing grade	Promega, Madison, WI, USA	Cat#V5111
Reprosil-Pur C18-AQ, 1.9 µm	Dr. Maisch, Ammerbuch, Germany	Cat#r13.aq
Reprosil-Pur 200 C18-AQ, 5 µm	Dr. Maisch, Ammerbuch, Germany	Cat#r13.aq
SDB-RPS membrane (Empore^TM^)	Merck Millipore, Darmstadt, Germany	Cat#2241
Digitonin	Sigma-Aldrich, St. Louis, MO, USA	Cat# D141
Cycloheximide	Sigma-Aldrich, St. Louis, MO, USA	Cat# C7698
Formic acid, MS grade	Thermo Fisher Scientific, Waltham, MA, USA	Cat# 85178
Cysteine	Sigma-Aldrich, St. Louis, MO, USA	Cat# C7352
INHIBITORS		
Protease Inhibitor Cocktail	Sigma-Aldrich, St. Louis, MO, USA	Cat#P8340
Phosphatase Inhibitor Cocktail 3	Sigma-Aldrich, St. Louis, MO, USA	Cat#P0044
Phosphatase Inhibitor Cocktail 2	Sigma-Aldrich, St. Louis, MO, USA	Cat#P5726
EXPERIMENTAL MODELS: ORGANISMS/STRAINS		
Wild type Xenopus laevis frogs	Nasco, Fort Atkinson, WI, Chicago, IL, USA	Cat#LM00456; RRID:XEP_Xla100
SOFTWARE AND DATABASES		
PEAKS Studio X+	Bioinformatics Solutions Inc., Waterloo, ON, Canada	Version 11
DIA-NN	Max Planck Institute of Biochemistry, Planegg, Germany	Version 2.2.0
UniProt Database (Xenopus laevis)	UniProt Consortium	Release March 2025
ProteomeXchange/PRIDE Repository	EMBL-EBI	Dataset PXD071213
ImageJ/Fiji	National Institutes of Health, Bethesda, MD, USA	Version 1.54f/Version 2.9.0
Python	Python Software Foundation	Version 3.12.7
pandas	PyData/NumFOCUS	Version 2.3.3
numpy	NumPy Developers	Version 2.3.4
seaborn	Michael Waskom	Version 0.13.2
R	R Foundation for Statistical Computing	Version 4.10.0
clusterProfiler	Guangchuang Yu	Version 4.10.0
Gene Ontology (GO) Database	Gene Ontology Consortium	Release 15 September 2024
KEGG Database	Kanehisa Laboratories	Release 27 May 2024
INSTRUMENTS		
Ultimate 3000 Nano LC System	Thermo Fisher Scientific, Waltham, MA, USA	N/A
Orbitrap Tribrid Lumos Mass Spectrometer	Thermo Fisher Scientific, Waltham, MA, USA	N/A
P-2000 Laser Puller	Sutter Instrument, Novato, CA, USA	N/A
Homemade analytical column	Self-packed; 300 × 0.1 mm, Reprosil-Pur C18-AQ, 1.9 µm.	N/A

## Data Availability

The mass spectrometry proteomics data have been deposited to the ProteomeXchange Consortium via the PRIDE partner repository with the dataset identifiers PXD071213 and PXD071283.

## Supplementary Materials

The following supporting information can be downloaded at: https://www.mdpi.com/article/10.3390/ijms27020738/s1.
