# Supplementary material for: Co-Immunoprecipitation-Coupled Mass Spectrometry Analysis of Zyxin’s Interactome and Phosphosites in Early Xenopus laevis Development
Source: Int J Mol Sci. 2026 Jan 11;27(2):738. doi: 10.3390/ijms27020738 (PMC12841194; doi:10.3390/ijms27020738)
Supplement: Supplementary file 1 [file ijms-27-00738-s001.zip › ijms-4036423-supplementary.pdf]

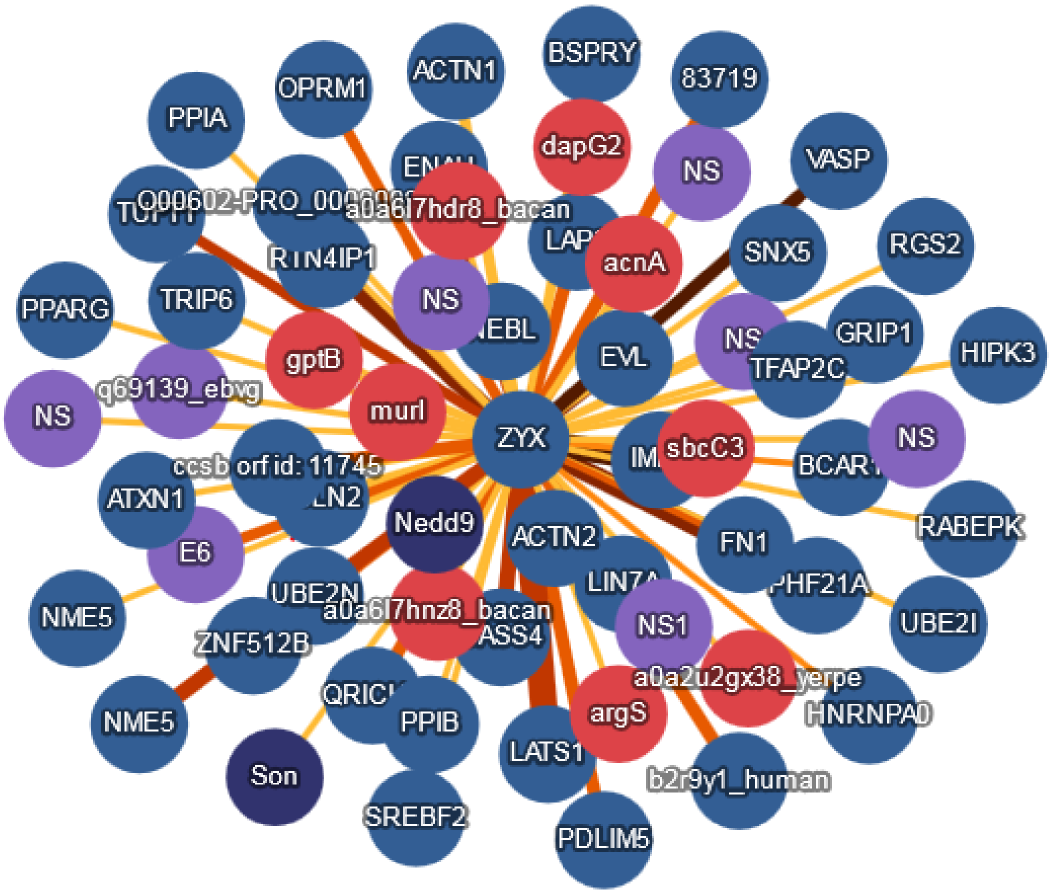

SUPPLEMENT, Figure S1.

Human ZYXIN (ZYX) interaction network showing experimentally validated and computationally predicted binding partners obtained from the IntAct molecular interaction database (https://www.ebi.ac.uk/intact). IntAct is an open-source, curated resource of molecular interaction data derived from the scientific literature and from direct user submissions.

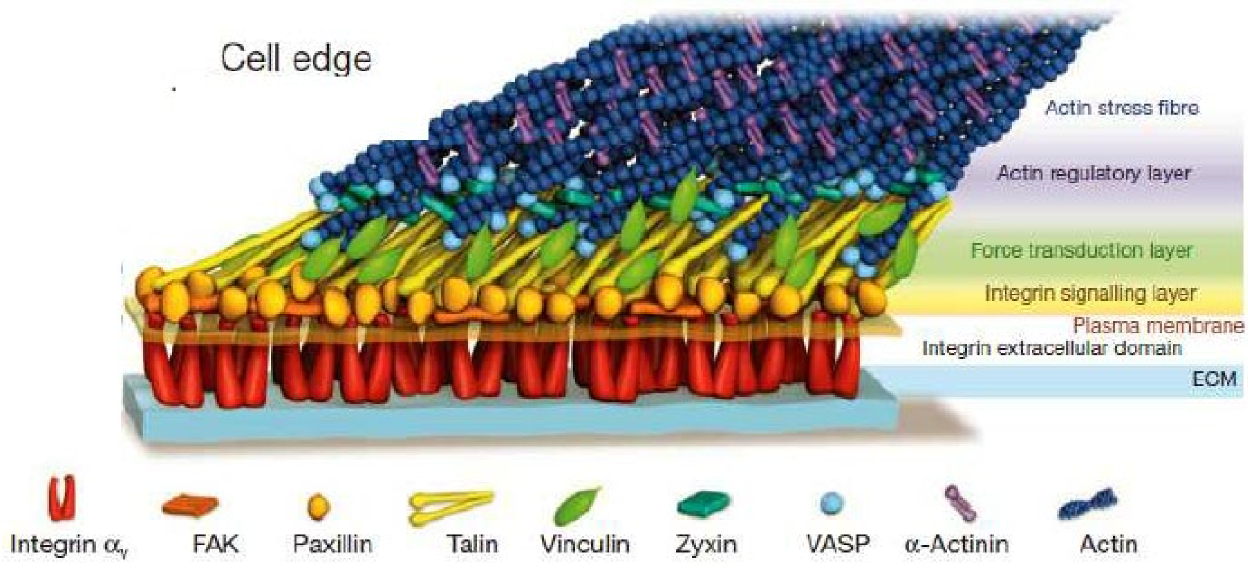

SUPPLEMENT, Figure S2. Molecular organization of integrin-based focal adhesions at the cell edge.

Integrins mediate cell attachment to the extracellular matrix, and their intracellular domains are linked to a stratified focal adhesion core comprising three functional layers: (1, yellow) a membrane-proximal integrin-signalling layer enriched in integrin cytoplasmic tails, focal adhesion kinase and paxillin that participate in signalling cascades controlling adhesion dynamics; (2, green) an intermediate force-transduction layer containing talin and vinculin that mechanically couple integrins to the actin cytoskeleton together with adaptor proteins such as zyxin and VASP; and (3, violet) an upper actin-regulatory layer organized primarily by actin filaments and α-actinin. Adapted from Kanchanawong et al., 2010 [52].

Protein	Gene	UniProt ID (Representative)	Detection (DDA/ DIA)	Stage Specificity	binding specificity	Notes
Talin 1	tln1	A0A8J1M208, Q6NRV2	Both	All stages	high	Core adhesion linker
Talin 2	tln2	A0A1L8G4N6, A0A974HNR6	Both	All stages	high	Core adhesion linker
Vinculin	vcl	A0A1L8FL43, P55312	Both	All stages	high	Core adhesion linker
α-Actinin 1	actn 1	A0A8J0TMH9, Q6NRW6	Both	All stages	very high	Actin cross-linker
α-Actinin 4	actn 4	A0A8J1LN98, A0A974H659	Both	All stages	very high	Actin cross-linker
β-Actin	actb	O93400	Both	All stages	average	Cytoskeletal polymer
Myosin-9	myh 9	A0A974HLZ4, O93522	Both	All stages (↑14)	high	Actin motor
Myosin-10	myo 10	A0A1L8GGP5, A0A8J1M4I9	Both	All stages (↑14)	high	Actin motor
VASP	vasp	A0A974H7T9, Q6GQB0	Both	All stages	very high	Actin polymerase
Integrin α-5	itga5	Q06274, A0A1L8HAA6	DIA	Stages 10, 14	low	ECM receptor
Integrin β-1	itgb1	P12606	DIA	Stages 10, 14	low	ECM receptor

SUPPLEMENT, Table S1**.** Core cytoskeletal and adhesome proteins co-immunoprecipitating with zyxin across early developmental stages in *Xenopus*.

The high-confidence interactors, detected by DDA and/or DIA mass spectrometry, include essential structural and regulatory components of focal adhesions and the actin cytoskeleton. The reported binding specificity is based on spectral counts and reproducibility across replicates.

Protein	Gene	UniProt ID	Identification Peptide(s) (Position)	Detection Method (Stage)	Inference / Note
Y-box protein 2	ybx2	Q920B5, A0A1L8FJH8	GAEAANVTGPGGVPVK (102-117); VLATQVQGCTVK 937-47)	DDA; DIA (Stages 10, 14)	Specific peptides identified
Y-box protein 1	ybx1/ ybx2	P16990, Q920B5	NGYGFINR (48-55); EDVFVHQTAIKK (60-71)<br><br>NDTKEDVFVHQTAIK (61-75 for Ybx2; 56-70 for Ybx1)<br>AGQEPAATVGEK (19-30 for Ybx1)	DDA; DIA (Stages 10, 14)<br><br>DIA (Stages 10, 14)	Via conserved signature peptides<br><br>Specific peptides identified
Giloblastoma 1	gli1	Q91690, A0A8J0UDR0	LGLLDGR (226-232)	DDA only (Stage 16)	Specific peptide identified

SUPPLEMENT, Table S2. Zyxin interactions with transcriptional regulators.

Proteins identified by data-dependent acquisition (DDA) and data-independent acquisition (DIA) are listed with the corresponding genes, UniProt identifiers, proteotypic peptides and embryonic stages of detection. Identification is supported either by specific peptides unique to the indicated protein or by conserved signature peptides that distinguish Ybx isoforms from one another.

**Domain / Peptide (Isoform)**	**Phosphosite(s)**	**Major Finding (Stage-Specificity)**	**Functional Implication**
N-terminal			
3P Peptide (A5H447)	pSer197/pSer198	High at stage 10, decreases to stage	Likely regulates "open" active conformation early in development.
4PPeptide (A0A8J1LC30)	pSer250	Consistently low at all stages	Isoform-specific low-level regulation.
Central Region			
Peptide 376-395 (Multiple)	pSer383,pSer383,pSer386	Present at stages 10 & 14	May be cell-cycle associated (mitosis).
LIM Domain 1			
Peptide 488-502 (A5H447)	pThr499 / pSer501	Very low stoichiometry<br>Present at stages 10 & 14	Novel finding. May modulate protein-binding capacity of LIM domain.

SUPPLEMENT, Table S3. Stage-specific phosphorylation dynamics of key zyxin peptides identified by DIA-MS in the Xenopus model.

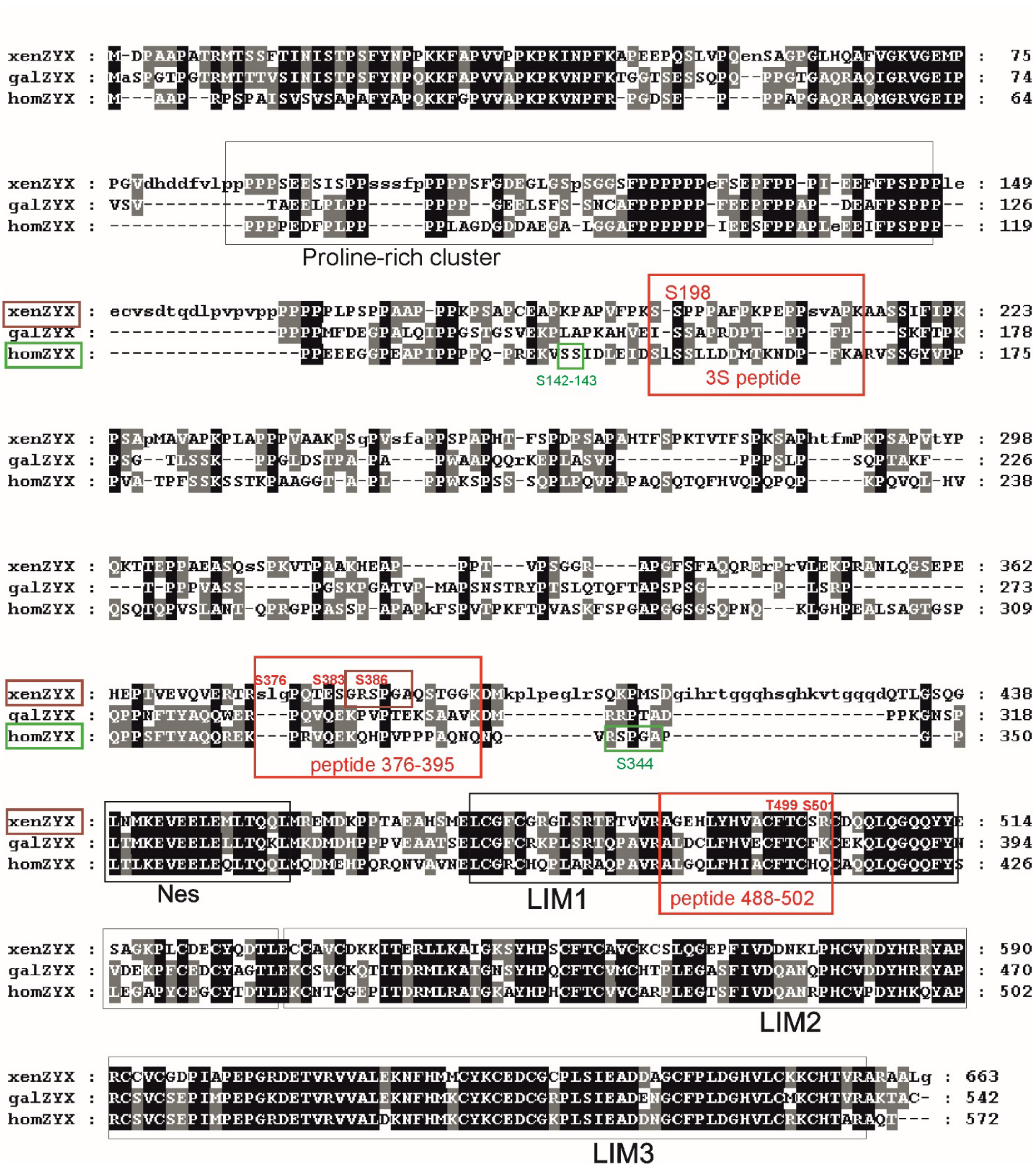

SUPPLEMENT, Figure S3. Mapping and comparative analysis of zyxin phosphorylation sites.

The main functional domains are outlined with black frames. Peptides identified as phosphorylated in the *Xenopus* model are highlighted in red. The aligned phosphosites and corresponding amino acid residues in human zyxin are annotated in green.

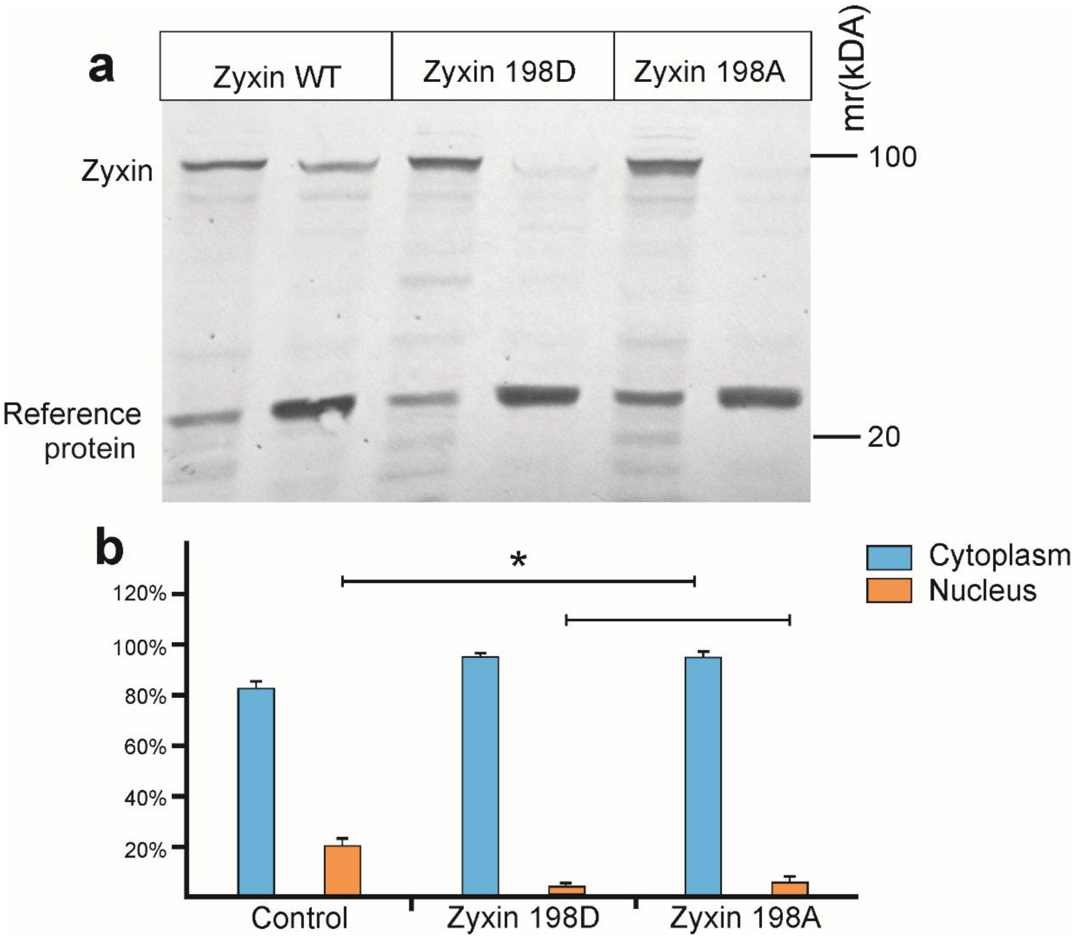

SUPPLEMENT, Figure S4. Subcellular localization of control and mutant Zyxin forms.

(a) Representative Western blot showing zyxin levels in cytoplasmic and nuclear fractions from Xenopus laevis embryos expressing control zyxin, zyxin 198A, and zyxin 198D. Loading control protein, was used as a loading control.
(b) Quantification of zyxin distribution between cytoplasm and nucleus. Data are presented as the mean percentage (±SEM) of the total cellular pool of the protein localized to each compartment (n=3). Statistical significance of differences in distribution between the control and mutant forms was determined by one-way ANOVA followed by Tukey's post hoc test (*$p < 0.05$).

Protein Category	Protein/ Isoform (Gene)	UniProt ID	Detection (Stage)	Proposed Functional Link to Zyxin
Kinase	AKT2 (akt2)	Q6IP76, Q7ZX15	DDA/DIA (10), DIA (14,16)	Potential kinase for N-terminal Ser197/198 (human Ser142 homolog).
	CDK1 (cdk1 a,b)	P35567, P24033	DDA (16)	Potential kinase for central Ser386 (mitotic phosphorylation).
	CDK13 (cdk13)	A0A1L8FWZ8	DDA/DIA Stages 14, 16	Function in development unclear; may regulate late-stage phosphorylation.
	CDK5 (cdk5)	P51166	DDA/DIA All stages, 14 max	early-expressed neuronal cyclin-dependent kinase
	CDK4 (cdk4)	Q91727	DIA (10, 16)	Probably involved in the control of the cell cycle.
Adapter/Scaffold	14-3-3β/ε/γ/ζ (ywhab/e/g/z)	Q5XHK2, Q6NRY9, Q91896, etc.	All stages	Binds phosphorylated zyxin; implicated in nuclear translocation and anti-apoptotic signaling [32].
Protease	Caspase-3.2 (casp3.2)	A0A1L8HLJ6, A0A8J0UPK1	All stages (high specificity)	Cleaves zyxin; links zyxin to apoptotic pathways.
Transcriptional Co-activator	Yap1 (yap1)	D6C652	Stages 14, 16	Potential for joint regulation of transcription; links cytoskeleton to nuclear signaling.

SUPPLEMENT, Table S4. Proteomic profiling of zyxin interactors.

The table lists key co-precipitating proteins implicated in phosphorylation, scaffolding, apoptosis, and transcription, providing a resource for understanding zyxin's stage-specific regulatory networks in development.

SUPPLEMENT, Table S5.

DIA method for overlapping windows. In the DIA, the MS scan events of experiments 1-4 were cycled.

DIA method: m/z500-740

Experiment 1	Full Scan (MS1 Scan)<br>Scan Range (m/z): 495-745
Experiment 2	DIA (MS2 Scan)<br>Isolation window m/zs: 1A
Experiment 3	Full Scan (MS1 Scan)<br>Scan Range (m/z): 495-745
Experiment 4	DIA (MS2 Scan)<br>Isolation window m/zs: 1B

**Scan**	**Isolation window m/zs 1A**	**Isolation window m/zs 1B**
1	498.4765-502.4783	500.4774-504.4792
2	502.4783-506.4801	504.4792-508.481
3	506.4801-510.4819	508.481-512.4828
4	510.4819-514.4837	512.4828-516.4847
5	514.4837-518.4856	516.4847-520.4865
6	518.4856-522.4874	520.4865-524.4883
7	522.4874-526.4892	524.4883-528.4901
8	526.4892-530.491	528.4901-532.4919
9	530.491-534.4928	532.4919-536.4937
10	534.4928-538.4947	536.4937-540.4956
11	538.4947-542.4965	540.4956-544.4974
12	542.4965-546.4983	544.4974-548.4992
13	546.4983-550.5001	548.4992-552.501
14	550.5001-554.5019	552.501-556.5028
15	554.5019-558.5038	556.5028-560.5047
16	558.5038-562.5056	560.5047-564.5065
17	562.5056-566.5074	564.5065-568.5083
18	566.5074-570.5092	568.5083-572.5101
19	570.5092-574.511	572.5101-576.5119
20	574.511-578.5128	576.5119-580.5138
21	578.5128-582.5147	580.5138-584.5156
22	582.5147-586.5165	584.5156-588.5174
23	586.5165-590.5183	588.5174-592.5192
24	590.5183-594.5201	592.5192-596.521
25	594.5201-598.5219	596.521-600.5229

26	598.5219-602.5238	600.5229-604.5247
27	602.5238-606.5256	604.5247-608.5265
28	606.5256-610.5274	608.5265-612.5283
29	610.5274-614.5292	612.5283-616.5301
30	614.5292-618.531	616.5301-620.5319
31	618.531-622.5329	620.5319-624.5338
32	622.5329-626.5347	624.5338-628.5356
33	626.5347-630.5365	628.5356-632.5374
34	630.5365-634.5383	632.5374-636.5392
35	634.5383-638.5401	636.5392-640.541
36	638.5401-642.5419	640.541-644.5429
37	642.5419-646.5438	644.5429-648.5447
38	646.5438-650.5456	648.5447-652.5465
39	650.5456-654.5474	652.5465-656.5483
40	654.5474-658.5492	656.5483-660.5501
41	658.5492-662.551	660.5501-664.552
42	662.551-666.5529	664.552-668.5538
43	666.5529-670.5547	668.5538-672.5556
44	670.5547-674.5565	672.5556-676.5574
45	674.5565-678.5583	676.5574-680.5592
46	678.5583-682.5601	680.5592-684.561
47	682.5601-686.562	684.561-688.5629
48	686.562-690.5638	688.5629-692.5647
49	690.5638-694.5656	692.5647-696.5665
50	694.5656-698.5674	696.5665-700.5683
51	698.5674-702.5692	700.5683-704.5701
52	702.5692-706.5711	704.5701-708.572
53	706.5711-710.5729	708.572-712.5738
54	710.5729-714.5747	712.5738-716.5756
55	714.5747-718.5765	716.5756-720.5774
56	718.5765-722.5783	720.5774-724.5792
57	722.5783-726.5801	724.5792-728.5811
58	726.5801-730.582	728.5811-732.5829
59	730.582-734.5838	732.5829-736.5847
60	734.5838-738.5856	736.5847-740.5865
61	738.5856-742.5874	
